# Laparoscopic hyperspectral imaging for in vivo detection of the vagal nerve in upper gastrointestinal surgery

**DOI:** 10.1007/s00464-025-12028-1

**Published:** 2025-08-11

**Authors:** Hannes Köhler, Annalena Ilgen, Annekatrin Pfahl, Sigmar Stelzner, Matthias Mehdorn, Boris Jansen‑Winkeln, Andreas Melzer, Ines Gockel, Yusef Moulla

**Affiliations:** 1 Innovation Center Computer Assisted Surgery (ICCAS), Faculty of Medicine, Leipzig University, Semmelweisstr. 14, 04103 Leipzig, Germany; 2 Department of Visceral, Transplant, Thoracic and Vascular Surgery, University Hospital of Leipzig, Leipzig, Germany; 3 Department of General, Visceral, Thoracic, and Vascular Surgery, Klinikum St. Georg, Leipzig, Germany; 4 School of Medicine, Institute for Medical Science and Technology (IMSaT), University of Dundee, Dundee, UK

**Keywords:** Hyperspectral imaging (HSI), Minimally invasive surgery (MIS), Vagal nerve, Machine learning

## Abstract

**Background:**

Accurate intraoperative detection of nerves in critical anatomic regions during oncologic resection, such as the vagal nerve, is crucial. The vagal nerve regulates many internal organs and is at risk during surgeries, potentially leading to severe complications. While different types of intraoperative neuromonitoring methods exist for functional assessment, Hyperspectral Imaging (HSI) offers a non-invasive, real-time alternative for morphologic identification. This study is, to our knowledge, the first to assess laparoscopic HSI for imaging and delineating the vagal nerve during minimally invasive surgery.

**Methods:**

This prospective cohort study examined the vagal nerves of 19 patients undergoing Ivor Lewis esophagectomy using a laparoscopic HSI system with a 30-degree optic during the thoracic part of the procedure. Measurements on the exposed vagal nerve collected spectral data from 500 to 995 nm at each pixel. Based on these reflectance spectra, four different state-of-the-art machine learning methods for binary and multi-class tissue differentiation were evaluated. The classifiers were validated using Leave-One-Patient-Out Cross-Validation (LOOCV) and k-fold CV.

**Results:**

The spectra of the tissue classes azygos vein, pleura, lung, vagal nerve, and esophagus showed high similarity, with wide inter-patient variability. All tested machine learning classifiers showed similar accuracy in differentiating vagal nerve tissue. Linear Discriminant Analysis (LDA) and Support Vector Machine (SVM) outperformed Logistic Regression (LR) and Multilayer Perceptron (MLP), with LDA showing the highest F1 score (harmonic mean of precision and recall) for binary classification (0.85), and SVM excelling in multi-class classification (0.74). Reflectance spectra without further pre-processing provided the best results for tissue differentiation.

**Conclusion:**

The first application of HSI to detect the vagal nerve during minimally invasive surgery has shown promising classification results for the five tissue classes considered and highlighted the technical challenges for clinical use. Further clinical research is needed to explore the full potential of HSI to improve the reliability of nerve classification during surgery.

Nerves are critical structures that need to be protected intraoperatively to prevent complications potentially causing motor and sensory deficits and consequently reducing patients’ quality of life. In some surgeries, detecting them intraoperatively with high precision is of utmost importance to maintain the nerve’s integrity and functionality. During minimally invasive surgery (MIS), surgeons have to rely on their visual knowledge to detect nerves or use other intraoperative methods to distinguish them from the surrounding tissue, such as intraoperative neuromonitoring (IONM).

The vagal nerve is known to be a sensitive structure during many surgeries, including thyroid, esophageal, and gastric procedures. It is the 10th cranial nerve and the main parasympathetic nerve that regulates the activity of many internal organs, including the pharynx, larynx, heart, lung, and the gastrointestinal tract until the so-called “Cannon-Boehm-Point,” parasympathically and general-viscerosensible. It carries sensory as well as motor fibers. Originating laterally from the medulla oblongata, the vagal nerve makes its way through the neck and the chest, where the recurrent laryngeal nerves (RLN) branch and innervate the larynx. The vagal nerve is closely attached to the esophagus and splits into an anterior and a posterior trunk, that enter the esophageal crura down to the abdomen.

Intraoperative damage to the vagal nerve is a feared complication and can lead to significant health issues and disruptions of normal body function. As one of the most extensive nerves, the vagal nerve plays a relevant role in controlling vital processes, making its impairment particularly concerning [1]. The consequences of the lesion depend on the location of the line break.

A lesion on its way through the neck (recurrent laryngeal nerve paralysis = RLNP), as it might occur during thyroidectomy, can lead—in the case of a one-sided impairment—to slight hoarseness, weak voice, a deterioration in the singing voice, and rapid vocal fatigue. More critically, bilateral injury to the recurrent laryngeal nerves can cause paralysis of both vocal cords, leading to severe breathing and swallowing difficulties. This condition may necessitate emergency airway management, including intubation or tracheostomy that can potentially be life-threatening [2].

Damages to the anterior and posterior vagal trunks can occur during benign esophageal or gastric surgery and are even mandatory from an oncologic point of view during esophagogastric resections due to cancer (except for vagal-spearing esophagectomy, the indications to which are rather rare). They can lead to motility disorders, delayed gastric emptying, this delayed gastric emptying may serve as a risk factor for the recurrence of gastroesophageal reflux disease (GERD) following hiatal hernia repair. Additionally, injury of vagal nerve can lead to secretion disorders of the stomach, especially to the (early- or late) dumping-syndrome, with e.g., counter-regulatory hypoglycemia [3, 4], and the described intestinal section, causing abdominal pain, constipation, hard and painful bowel movements, and even a functional ileus [5]. Furthermore, the impairment can result in malassimilation with chronic diarrhea, electrolyte imbalance, and weight loss [6].

To prevent these far-reaching consequences of a vagal impairment, intraoperative monitoring of the vagal nerve is always an important aim, except for oncologic esophagogastric surgery, where the anterior and posterior trunc of the nerve have to be divided inevitably. Several different methods have been investigated so far, some of which are non-invasive, like Raman spectroscopy [7, 8], spectroscopy [9–13], polarimetric imaging [14–16], and autofluorescence imaging [17]. Others are invasive, like dye-based methods [18, 19] or using electric impulses to monitor the nerve’s integrity and functionality (neuromonitoring). However, only the latter method is currently used in clinical practice with an emphasis on thyroidectomy procedures with intraoperative (continuous) monitoring of the RLN, and rectal surgery, monitoring the pelvic nerves [20].

Hyperspectral Imaging (HSI) represents a non-invasive, contactless method with high innovative potential, offering real-time intraoperative insights into the nature and characteristics of the current tissue within a matter of seconds [21]. The intraoperative Region Of Interest (ROI) is exposed to visible and near-infrared light, and subsequent measurement of the light remitted by the tissue is performed [22]. This approach extends the information available to surgeons beyond the capabilities of the human eye, providing valuable details about the tissue and the diverse structures under consideration during surgical procedures [23]. Presently, its application includes identifying optimal regions for resection borders in cases of gastrointestinal anastomoses and guiding in liver resections [24, 25]. Two in vivo studies investigated HSI for the detection of the RLN in humans and pigs [26, 27]. Furthermore, ratiometric approaches for contrast enhancement of nerves in humans, animals, and cadaver models have been reported [12, 13, 28].

The objective of our current investigation was to assess the suitability of the new laparoscopic HSI system TIVITA® Mini for accurately imaging and delineating the vagal nerve in the context of minimally invasive esophageal surgery. This evaluation is intended to explore its potential application in other minimally invasive procedures, such as anti-reflux surgery, where preserving the integrity of the nerve is critical.

## Materials and methods

### Patients

In this prospective cohort study, the vagal nerves of 19 patients (aged 18 and above) were measured. Patients underwent one-time Ivor Lewis esophagectomy due to adenocarcinomas of the esophagogastric junction (EGJ), squamous cell carcinomas of the esophagus, or perforation at the University Hospital of Leipzig between January 2023 and July 2023. A hyperspectral intraoperative imaging system was used to detect the vagal nerve during surgery, as described below in detail. Patients unable to consent or being pregnant were excluded from this study.

Follow-up outcome data, encompassing early morbidity and mortality within the initial 30 days post-surgery, were gathered and analyzed. Subsequent postoperative evaluations adhered to established protocols.

### Surgical procedure and hyperspectral imaging

**Fig. 1:**
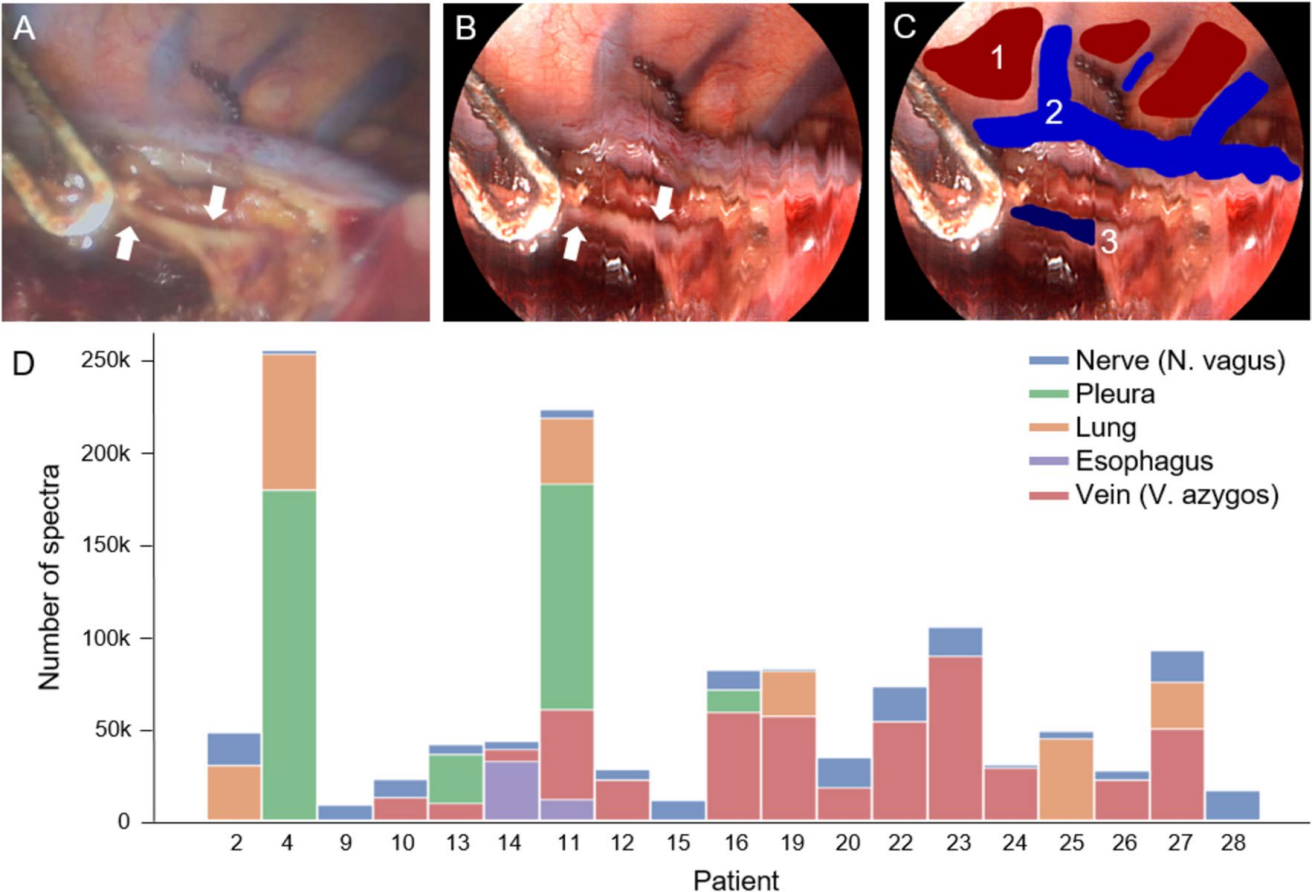
Intraoperatively acquired image data. **A** Snapshot provided by the integrated color image sensor during HSI record. The two white arrows point at the vagal nerve. **B** Reconstructed color image based on hyperspectral data. The respective anatomical positions from **A** are labeled with white arrows. **C** Ground truth mask after manual annotation of the pleura (1), azygos vein (2), and vagal nerve (3) used to select the corresponding spectra for the machine learning tasks. **D** Statistical distribution of the acquired spectra (pixel) per patient and tissue class

All oncologic esophagectomies were conducted as Ivor Lewis procedures and adhered to established standard protocols. During the thoracic part, when the vagal nerve was carefully exposed macroscopically and assessed prior to its transection in the region of the anastomosis, the TIVITA® Mini system (Diaspective Vision GmbH, Am Salzhaff-Pepelow, Germany) was used to perform the measurements. This device is a medically approved HSI system designed for MIS and has a 30-degree laparoscopic optic from Karl Storz SE & Co. KG (Tuttlingen, Germany). It operates as a push-broom scanning HSI system, analyzing the wavelength-specific reflectance of an object at each pixel of the captured image. The system covers a wavelength range from 500 to 995 nm with high spectral resolution in 5 nm increments, resulting in one hundred spectral bands. This functionality enables the analysis of distinct spectral reflectance properties of various tissues, providing the surgeon with information regarding the tissue oxygenation of the superficial tissue layer (StO2 in %). For the deeper tissue layers (4–6 mm), the system utilizes a Near-Infrared Perfusion Index (NIR-PI) ranging from 0 to 100 to estimate tissue oxygenation. Additionally, the Organ Hemoglobin Index (OHI from 0 to 100) offers insights into the distribution of hemoglobin, while the Tissue Water Index (TWI from 0 to 100) represents the water content of the observed tissue. Furthermore, a color image can be reconstructed from the spectral data, and a high-resolution video is provided by an integrated color image sensor as shown in Fig. 1A, B.

In this study, switching on and preparation of the TIVITA® Mini system began several minutes before the measurement, employing a sterile plastic cover to minimize the time loss during the surgical procedure. The minimally invasive HSI optic was inserted through the assistant trocar during the thoracoscopic (conventionally minimally invasive or robotic) phase of the oncologic esophagectomy to measure the vagal nerve. For this purpose, the vagal nerve was exposed and measured during the mobilization of the esophagus, but prior to its transection. Before the measurement, the nerve and the surrounding area underwent irrigation to minimize artifacts arising from the fresh or coagulated blood and to maximize the exposure of the target structure. All measurements of the vagal nerve were conducted in situ at a standardized distance of five to seven centimeters (from the tip of the optical system to the target structure, the vagal nerve). Additionally, the regular laparoscopic/thoracoscopic or robotic optic was removed from the situs and the operating room lighting was turned off during data acquisition to reduce potential artifacts caused by external illumination. Since performing one real-time measurement only takes about five seconds, two to three measurements were always possible without prolonging the surgery substantially. During the measurement process, the laparoscope was kept as still as possible to minimize movement artifacts.

### Data analysis and machine learning

In total, 25 measurements in 19 patients were included. Due to spectral artifacts, the recordings of one patient had to be excluded. After labeling by medical experts, the data set included reflectance spectra of five different tissue classes: azygos vein (480.541), pleura (341.391), lung (233.548), vagal nerve (178.241), and esophagus (44.462). An example of a reconstructed color image from an HSI record after manual annotation of three tissue classes is shown in Fig. 1C.

Four spectral pre-processing methods were compared after spectral and spatial Gaussian smoothing (σ = 2) of the HSI data. These were the use of the reflectance, Standard Normal Variate (SNV), first derivative, and second derivative of the reflectance spectra.

Two pixel-level classification tasks were investigated: binary classification of spectra from the vagal nerve against the other four tissue types merged as one class (task 1) and multi-class differentiation of all five tissue types (task 2). Four different state-of-the-art machine learning methods: Support Vector Machine (SVM), Linear Discriminant Analysis (LDA), Logistic Regression (LR), and Multilayer Perceptron (MLP), were evaluated for classification tasks 1 (binary) and 2 (multi-class). The manual labeling of the images by medical experts served as ground truth.

The four classifiers were validated by Leave-One-Patient-Out Cross-Validation (LOOCV) and repeated stratified k-fold Cross-Validation (k-fold CV) with ten folds and three repeats in task 1, while only LOOCV was used for task 2. The dataset is unbalanced in terms of the number of spectra per tissue class overall and per patient, as can be seen in Fig. 1D. Therefore, random undersampling of the classes was performed such that the number of spectra did not exceed that in the nerve class. This was used for data balancing of the training and test data in k-fold CV, while in LOOCV only for the training data.

The used evaluation metrics were accuracy, F1 score (weighted), precision, recall (sensitivity), and AUC score. The F1 score is a metric commonly used in statistics and machine learning to evaluate the accuracy of a test, particularly in classification tasks. It is the harmonic mean of precision and recall and gives a single metric that balances the trade-off between both (1). In diagnostic contexts, F1 score is valuable when there is an imbalance in classes.

1 $$F1 score=2 \frac{precision\times recall}{precision+recall}=\frac{2 TP}{2 TP+FP+FN}$$

For task 2 the weighted average of precision and recall over all classes was used. Color maps were superimposed over the reconstructed color images to visualize the classification confidence achieved in LOOCV in task 1.

Python 3.6 with scikit-learn 0.24.2 and Seaborn 0.11.2 library were used for data processing and data visualization, respectively.

## Results

### Patients’ characteristics

The study comprises 19 patients who underwent oncologic esophagectomies or esophagectomy due to esophageal perforation (n = 1). The surgical procedures included the robotic-assisted minimally invasive esophagectomy (RAMIE) in nine patients, the traditional minimally invasive esophagectomy (TMIE) in seven patients, a hybrid approach (laparoscopic abdominal part, open thoracic part) in two patients, and one complete open esophagectomy with gastric tube pull-up and cervical esophago-gastrostomy. All patients, except for one (= perforation), had a histopathologically confirmed diagnosis of esophageal carcinoma or carcinoma of the esophagogastric junction, with fifteen cases of adenocarcinoma and three cases of squamous cell carcinoma. The average age of the patients was 61.3 (± 8.1) years, with an average body mass index (BMI) of 27.4 (± 5.9) kg/m^2^. Detailed patient characteristics are delineated in Table 1.

**Table 1:** Preoperative findings and demographic data

Patient characteristics	N = 19
Sex, n (males:females)	15:4
Age (mean years ± SD)	61.2 ± 8.4
ASA classification	
ASA I	0
ASA II	11
ASA III	7
ASA IV	1
BMI (mean ± SD)	28.4 ± 5.4
Underweight (BMI: < 18.5)	0
Normal weight (BMI: 18.5 to 24.9)	7
Overweight (BMI: 25 to 29.9)	5
Obesity grade I (BMI: 30 to 34.9)	4
Obesity grade II (BMI: 35 to 39.9)	3
Obesity grade III (BMI: ≥ 40)	0
Diabetes mellitus	5
Arterial hypertension	9
COPD	1
Smoking	4
Histopathological entity	
Adenocarcinoma	15
Squamous cell carcinoma	3
No carcinoma (perforation)	1

*COPD* Chronic obstructive pulmonary disease

None of the patients suffered from postoperative anastomotic insufficiency. Postoperative pneumonia occurred in one case. Further intra- and postoperative findings can be found in Table 2.

**Table 2:** Intra- and postoperative findings and follow-up

Patient characteristics	N = 19
Type of operation	
RAMIE (Robotic-Assisted Minimally Invasive Esophagectomy)	9
TMIE (Total Minimally Invasive Esophagectomy)	7
HE (Hybrid Esophagectomy)	2
OE (open Esophagectomy)	1
Duration of surgery	
< 360 min	14
> 360 min	5
UICC classification	
0	2
I	3
II	2
III	11
IV	1
Postoperative pneumonia	1
Length of hospital stay (days) (median {range})	13 {9; 115}

*UICC * Union Internationale Contre le Cancer

### Classification results

Quantitative results for the binary (task 1) and multi-class (task 2) classification obtained using LOOCV and k-fold CV for evaluation of the classifiers LDA, LR, MLP, and SVM are given in Table 3. Using the reflectance yielded the best results for differentiating between the vagal nerve and the remaining tissue, regardless of the classification method used (except for the MLP in LOOCV, where the second derivative resulted in the highest scores). LDA resulted in the highest accuracy (84%), F1 score (85%), and precision (45%) in task 1. However, the use of SVM in combination with reflectance in task 2 outperformed the other classifiers on all observed metrics (74%). Using the k-fold CV for the evaluation of task 1, overfitting is more likely than with LOOCV because spectral data of one patient can be part of the training and test data sets. This effect can be seen in higher F1 scores, especially for the MLP in combination with reflectance (99%). This confirms the relevance of LOOCV for the most realistic assessment of classification performance to avoid overfitting when using medical spectral image data.

**Table 3:** Classification results compared to human annotation for task 1 (binary) and task 2 (multi-class)

Method	Accuracy LOOCV	F1 score (weighted) LOOCV/ k-fold CV	Precision LOOCV	Recall LOOCV	Best pre-proc LOOCV/ k-fold CV
Task 1 (Nerve vs. Other)					
LDA	**0.835**	**0.852**/ 0.871	**0.447**	0.762	Refl
LR	0.823	0.842/ 0.875	0.429	**0.813**	Refl
MLP	0.832	0.850/ **0.992**	0.442	0.772	2nd deriv./ Refl
SVM	0.816	0.838/ 0.880	0.418	0.805	Refl
Task 2 (5 tissue classes)					
LDA	0.669	0.675/ -	0.710	0.669	1st. deriv
LR	0.686	0.694/ -	0.723	0.686	SNV
MLP	0.630	0.612/ -	0.660	0.630	Refl
SVM	**0.735**	**0.735**/ -	**0.745**	**0.735**	Refl

The highest scores for each task are represented in bold

The scores and the best pre-processing technique are given for each of the four methods tested. The highest scores for each task are printed in bold

**Fig. 2:**
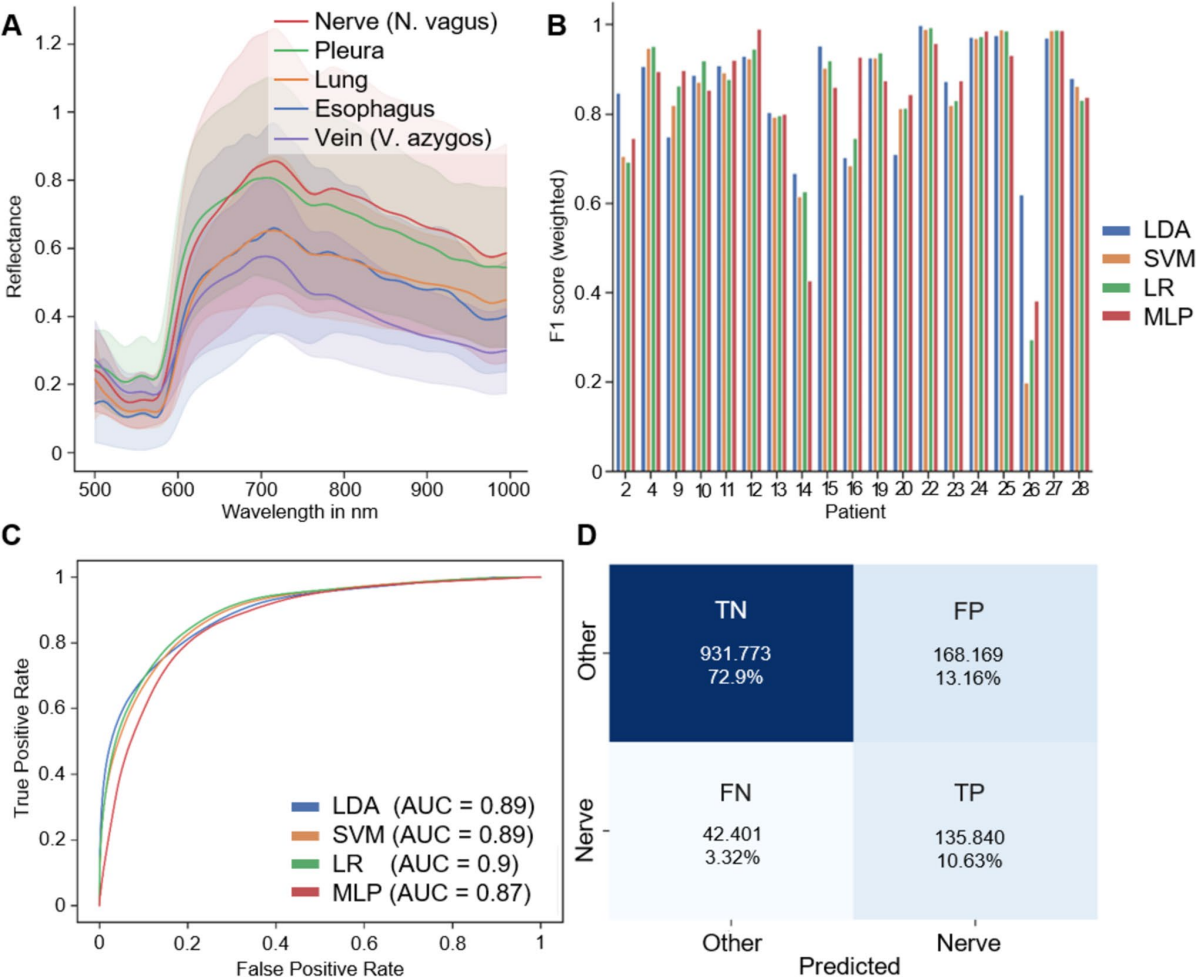
Quantitative results obtained using reflectance and LOOCV for classification task 1. **A** Mean reflectances of the five tissue classes over all patients. Standard deviations are shown as colored areas. **B** F1 score for each patient and classification method. **C** Receiver operating characteristic curve (ROC curve) and area under the curve (AUC) for the investigated classifiers.** D** Confusion matrix based on the number of spectra tested during LOOCV with LDA

The analysis of spectral differences between patients and organs showed a high similarity between the five tissue classes considered, with high inter-patient variability. Figure 2A shows the mean reflectances of the five tissue classes across all patients and the corresponding standard deviations from 500 to 995 nm. The obtained F1 scores using reflectance and LOOCV for classification task 1 are depicted in Fig. 2B for each patient and classification method. Except for three patients (14, 16, and 26), the classifiers showed similar results, which is also reflected in the ROC curves (Fig. 2C). The confusion matrix in Fig. 2D reveals a high number of false positives, which is the reason for the poor precision values of all classification methods in LOOCV. This is due to the low number of spectra in the Nerve class compared to the Other class in the LOOCV test data, as these are not balanced in contrast to the training data. A more favorable ratio is apparent in task 2 (multi-class), which enables improved precision values but reduced recall.

**Fig. 3:**
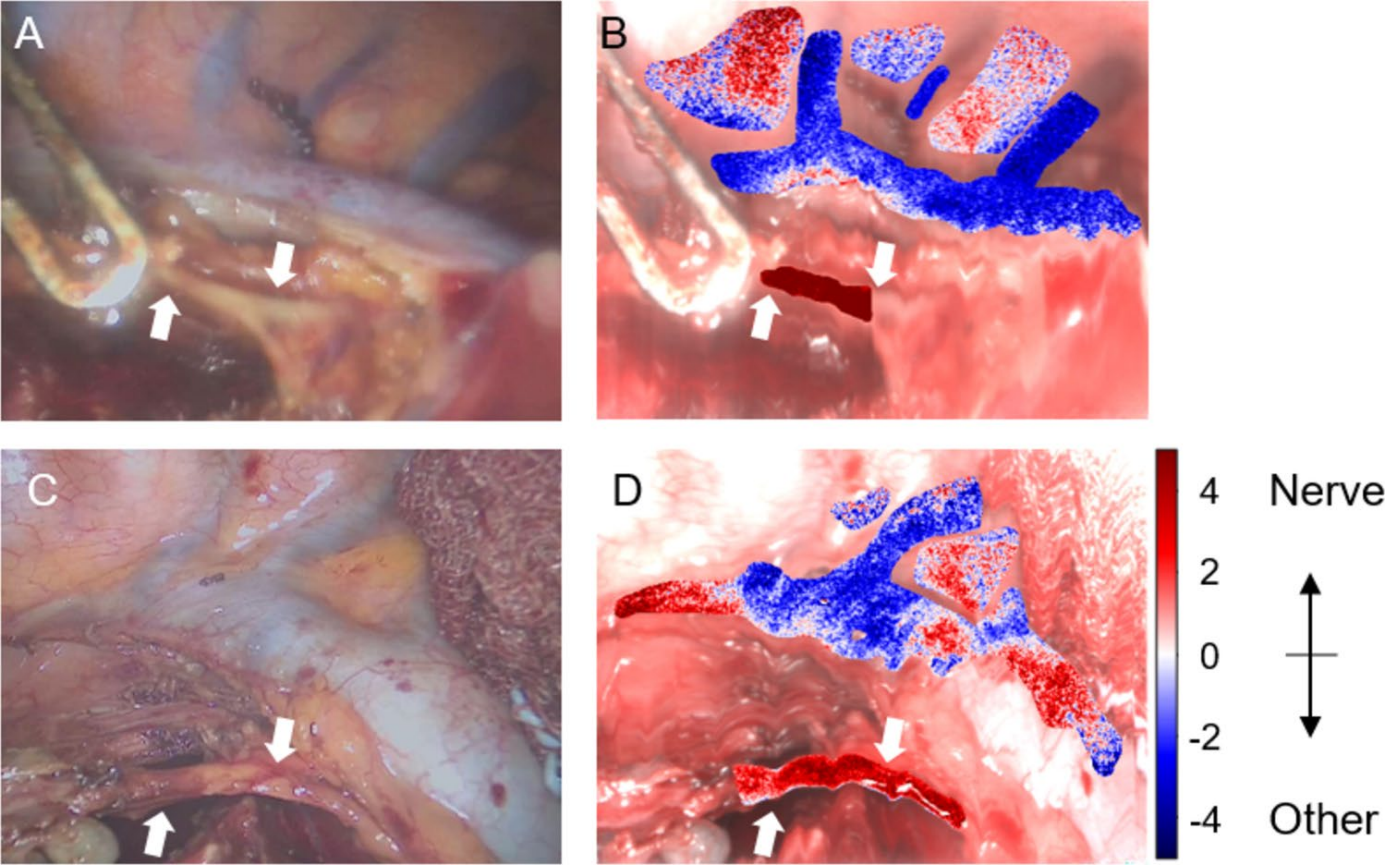
Visualization of the binary classification results using reflectance and LDA. **A, C** Color image during HSI record in patients 11 and 16. The vagal nerve is labeled with white arrows. **B, D** Confidence maps of the classification probability are superimposed on the reconstructed color image from HSI data. Positive values indicate the prediction as nerve tissue, while negative values represent the remaining tissue class. The used LDA models were trained without data from patients 11 and 16, respectively

To illustrate the classification obtained from two HSI records with moderate results (patients 11 and 16), Fig. 3 shows the confidence maps superimposed on the reconstructed color images. Some pixels in the area of the pleura and the azygos vein are incorrectly assigned to the nerve class, which contributes to the low precision. The area of the nerve contains mostly correctly classified pixels, which leads to good recall.

## Discussion

Hyperspectral imaging is a non-invasive, contact-free intraoperative method currently utilized for the identification of perfusion parameters in gastrointestinal surgery, primarily in the context of prediction of undisturbed anastomotic healing. The advancement of HSI systems for application in MIS has opened up a new and vast potential field. Structures that were previously inaccessible or lacked suitable monitoring methods in minimally invasive settings are now reachable and made visible.

An example of such structures includes neuronal tissues such as nerves, which are vulnerable to injury during any surgical procedure or intervention. In some cases, vagal nerve injury, e.g., during laparoscopic anti-reflux surgery, can lead to delayed gastric emptying, pyloric spasm, dumping, and to recurrence of gastroesophageal reflux. Therefore, protecting such sensitive structures is of paramount importance to maintain patients’ quality of life and to achieve better postoperative outcomes [29].

Various methods exist to ensure such protection. One method is IONM, which encompasses the continuous assessment of neurological signals, including electromyography (EMG), evoked potentials, and nerve stimulations, aiming at averting potential injury or dysfunction arising from surgical maneuvers or intraoperative trauma. While particularly advantageous in intricate surgical procedures involving neurological structures, its integration into minimally invasive surgeries remains pending. Electrophysiological nerve monitoring requires direct contact, the placement of electrodes, and often repeated stimulation to confirm nerve identity. It can be prone to false negatives, adds complexity, and does not provide continuous visual feedback [30].

Another method is the invasive, contrast agent-based method using Indocyanine Green (ICG), which reacts to nerves and, thus, enhances their visualization. This procedure is also feasible in the minimally invasive setting. However, there are some disadvantages, such as limited depth perception, false-positive results due to fluorescence of other tissues, temporal dependence on injection, and the use of contrast agents, which may entail possible allergic or cardiovascular reactions [31].

In contrast, HSI is non-contact, label-free, and does not rely on anatomical consistency or nerve stimulation. It allows for continuous real-time visualization without interrupting the surgical flow, and is compatible with both open and minimally invasive settings.

Among the non-invasive optical methods not yet integrated into clinical practice are those described above: Raman spectroscopy, spectroscopy, polarimetric- and autofluorescence imaging. Like HSI, these methods have the advantage of being contact-free and validated repeatable.

Previous work by Schols et al. [9] using diffuse reflectance spectroscopy for the differentiation between nerve and adipose tissue showed that the spectral range from 1000 to 1210 nm is more suitable than the spectral range available with the laparoscopic HSI system used in our work. Two other groups confirmed these findings in human cadavers and attributed this to the reduction in fat content and the increase in water and collagen content in the fascicular nerve tissue that can be observed between 1200 and 1600 nm [12, 32, 33]. Langhout et al. [10] compared nerve and surrounding tissue from humans and swine in vivo and post mortem. The use of human in vivo data for classification showed similar results to ours. Transfer learning based on additional animal and ex vivo data showed no improvement and limited transferability between the models [10]. Furthermore, spectral data (350 to 1830 nm) from ex vivo porcine nerves were claimed insufficient as training data for in vivo human nerve classification. Therefore, the use of spectral imaging for the acquisition of clinical data was suggested [11].

However, to the best of our knowledge, there are currently no laparoscopic systems for spectral imaging above 1000 nm. Besides the already mentioned work on contrast enhancement, spectral imaging has only been investigated below 1000 nm for nerve detection in open surgery. In a pilot study by Maktabi et al. [26], the TIVITA® Tissue system was used in vivo to detect the RLN in four patients. Even though the classification results were poor due to the small number of patients, a lower reflectance of the nerves compared to the surrounding tissue between 800 and 960 nm was observed, again indicating an increased water content. Barberio et al. used the same device and nerve structure in eight pigs [27]. Compared to our results, they achieved a similar sensitivity (0.76) but a higher AUC score (0.99) for the nerve class employing a 3D convolutional neural network (CNN) with LOOCV.

In contrast to several previous studies on other nerve structures, no increased water content of the vagal nerve was observed based on the reflectance spectra in our current analyses.

Besides the small number of patients included, the study has some limitations, mostly due to the technical challenges of data acquisition. As previously reported by our group, some of the HSI records show severe motion artifacts due to the movement of the tissue when the images were taken in minimally invasive thoracic procedures [34]. Due to these movement artifacts, full semantic annotation of all tissue structures in an image was unfortunately not possible. Therefore, the number of tissue types used is limited and the morphological information of an image is currently not used for tissue classification. In addition to the challenges of laparoscopic data acquisition, this might have contributed to the lower AUC score as compared to Barberio et al. [27]. Motion artifact compensation would not only eliminate the two limitations mentioned but would also enable a direct comparison between classification methods based on HSI data and recordings from the color image sensor.

Although subsurface detection was not demonstrated here, this work represents a foundational step toward that application. Additionally, even when the nerve is exposed, its identification can be challenging in cases of scarring, inflammation, or variant anatomy.

In conclusion, the first use of HSI for vagal nerve detection during MIS showed promising classification results for the differentiation of the tissue classes considered and revealed the technical challenges for the clinical application of this method. The system was able to accurately identify the nerve between 74 and 85% of the time, depending on the classification task. Our results are already comparable to previous work under more controlled conditions in open surgery. However, for the reliable classification of narrow nerve structures, the full capabilities of HSI in terms of the spectral range used, spatial information, penetration depth, and polarization should be investigated in further clinical studies. Upcoming studies will include lymphatic structures, such as the thoracic duct, which can be visually mistaken for nerves in surgical fields. These steps will advance the clinical applicability of HSI for reliable, non-invasive nerve identification.
